# Standards of care in neuromuscular fields

**DOI:** 10.1186/1471-2474-14-S2-O12

**Published:** 2013-05-29

**Authors:** Volker Straub

**Affiliations:** 1 Institute of Genetic Medicine, Newcastle University, International Centre for Life, Newcastle upon Tyne, UK

## 

There is general consensus that consensus guidelines for care standards are an important tool to provide both healthcare professionals and the patient community with up to date information about all aspects of best practices in a specific disease area. The specific development of care guidelines for neuromuscular diseases poses a number of challenges. All neuromuscular diseases are rare and many show a broad spectrum of clinical and genetic heterogeneity. A paucity of randomized controlled trials for most conditions means that best-practice care guidelines are often non-existent or poorly developed. Where they exist, healthcare professionals may be unaware of them. In many cases the lack of care guidelines contributes to a lack of clinical trial activity, as the implementation of care guidelines is also a prerequisite for a reliable comparison of outcome measures in clinical trials, especially in the neuromuscular disease field, where trials are normally multi-centric and international. Over the past years there have been various collaborative efforts to achieve trial readiness for a number of neuromuscular diseases by establishing care guidelines, patient registries and networks of care and trial sites. For some of the most common neuromuscular diseases like spinal muscular atrophy (SMA) and Duchenne muscular dystrophy (DMD) there are now international care guidelines for healthcare professionals and patients and families. Key factors that have helped to establish these care guidelines were the involvement of patient organizations, translational research networks, and health agencies and combining existing evidence with consensus among a large number of experts in different care areas. Once care guidelines have been established, it is important to disseminate them to the stakeholder community through clinics, meetings, journal and website publications, media interviews, patient registries and professional training courses. It is also crucial to keep care guidelines updated and to take new therapeutic developments and health research outcomes into account. Once care guidelines have been agreed and disseminated, it then becomes important to monitor their implementation and to assess whether they do indeed have a positive impact on health. This is particularly important for guidelines that are based on expert opinions that lack higher levels of evidence. Finally, we need to better understand why, despite agreed upon care guidelines for various neuromuscular diseases, many patients do not receive the treatment they describe.

